# An urgent call for action: Lebanon's children are falling through the cracks after economic collapse and a destructive blast

**DOI:** 10.1017/gmh.2022.14

**Published:** 2022-04-07

**Authors:** Rita Obeid, Sabine Saade

**Affiliations:** 1 Case Western Reserve University, Cleveland, Ohio; 2 The American University of Beirut, Beirut, Lebanon

“The true measure of a nation's standing is how well it attends to its children – their health and safety, their material security, their education and socialization, and their sense of being loved, valued, and included in the families and societies into which they are born.” (UNICEF, 2007).

In Lebanon, people are currently experiencing what has been called one of the top three most severe economic crises the world has seen since the mid-nineteenth century (World Bank Group, 2021). Since October 2019, the economic situation in Lebanon has been rapidly deteriorating (Youssef, 2020). In addition to the economic collapse, on 4 August 2020, a massive explosion at the Port of Beirut killed at least 200 people, injured thousands, and left over 300 000 people homeless (Devi, 2020). The explosion was so massive that it was felt 150 miles away in the neighboring island of Cyprus (Alberti, 2020). Since then, Lebanon has continued to experience economic downfall. To date, the Lebanese Lira has lost over 90% of its value (Associated Press, 2022). Since October 2019, the exchange rate of the US dollar went from 1500 Lebanese pounds (LBP) to 23 000 LBP, a rate that is constantly changing. To date, the minimum monthly wage in Lebanon remains 675 000 LBP, once worth $450 now averages around $30. With inflation up by 145%, families now spend up to five times the minimum wage on food alone (Goyeneche and Khraiche, 2021; Ramadan, 2021). To put things in perspective, 1 liter of milk (i.e. 0.33 fl oz) used to cost 3000 LBP before the inflation, this same liter of milk now costs 22 000 LBP (Hussein, 2021). Note that prices of goods are constantly changing. The country is also experiencing scarcity in a number of basic necessities (Goyeneche and Khraiche, 2021). Amongst those necessities are fuel, electricity, medication, medical services, milk, and infant formula. Furthermore, the COVID-19 pandemic and the lockdowns imposed as a result, exacerbated the challenges Lebanon is facing. The strain on the healthcare systems has been especially drastic for a population living in a country that is already on the brink of bankruptcy (Bizri *et al*., 2021). Measures taken to control the spread of the pandemic have also negatively impacted the education sector in Lebanon, in addition to putting a strain on the psychological health of its population (Bizri *et al*., 2021). For a country facing economic and political meltdown, the pandemic could not have come at a worse time (Bizri *et al*., 2021).

Following the banking crisis and the port explosion, a large number of highly trained individuals, including physicians, psychologists, and academics, have been seeking opportunities abroad (Vohra, 2021). Lebanon, once a hub of healthcare and higher education in the region, is experiencing ‘brain drain’ that will likely impact social cohesion and stability for the long term (Ben Brik, 2021). While the crisis has caught the attention of international newspapers and journals (Devi, 2020), one vulnerable population is, for the most part, left out of this ‘spotlight’: Lebanon's children. In particular, children with disabilities have been especially affected by these upheavals. Lebanese children's health and safety needs are no longer adequately met. Between the skyrocketing costs associated with inflation, and the loss of trained personnel, early intervention services (which are much needed for many children with disabilities to thrive) have become a rare luxury only the affluent few can afford.

Focusing on Lebanon's children is a pressing matter. Between the years 2019 and 2020, the poverty rate in Lebanon doubled to 55% and extreme poverty rose to 28%. Extreme poverty is defined as living on less than $1.90 per day (World Bank, n.d.). As such, 82% of families are now being described as multidimensionally poor, deprived of several of the following: Education, electricity, healthcare, medicine, employment, and/or assets (ESCWA, 2021). Currently, 80% of children in Lebanon are worse off than they were at the beginning of the year 2020. In a very short period, many families went from being categorized as middle class to falling below the poverty line and having great difficulty providing basic necessities for their children (ESCWA, 2020).

Academic, cognitive, and psychosocial problems experienced by children exposed to extreme poverty are well documented in the literature (Alaimo *et al*., 2001; Hair *et al*., 2015; Najman *et al*., 2009). Such difficulties are exacerbated for children with developmental delays and disorders (De los Reyes-Aragon *et al*., 2016). The literature on the efficacy and timeliness of early intervention is robust (e.g., Landa, 2018) with a push toward focusing on combating disparities and making sure that children from low-income backgrounds receive the services they need to thrive (De los Reyes-Aragon *et al*., 2016). Only a few studies assess the prevalence rates for developmental disabilities in Lebanon (Autism Spectrum Disorder [ASD]: 1.5%; Chaaya et al., 2016; and Attention Deficit Hyperactivity Disorder [ADHD]: 3.2%; Richa et al., 2014). These studies are scarce and their methodologies vary. While early intervention services are available in Lebanon, there is a shortage of qualified individuals who can provide such services (Naal *et al*., 2021). The treatment gap for mental health problems in Lebanon was estimated at over 90% (not specifically pertaining to children with disabilities; Shehadeh *et al*., 2020). The economic and political crisis Lebanon is currently experiencing, coupled with the COVID-19 outbreak, may further exacerbate this shortage, leaving thousands of vulnerable children without sufficient interventions. Additionally, the crisis has made such services unaffordable for parents of children with developmental delays and disorders.

## Possible solutions and paths for support

In light of this bleak situation and the importance of early intervention services, the need for immediate action is heightened (UNICEF Report, 2021). This next section highlights some possible avenues to support the children of Lebanon.

### Community-wide and one-to-one interventions

In situations where a community as a whole is experiencing a crisis, the need to shift from individualized therapeutic interventions to a more collective approach toward mental health has been suggested as a possible solution (Bosqui, 2020). While approaches focusing on the individual are useful in the West, the need for community-wide, culturally sensitive interventions becomes critical when an entire population is experiencing a crisis (Bosqui, 2020). Although not adequate for all individuals with developmental delays and disorders, group-delivered interventions could represent a more affordable and accessible option than one-to-one interventions (e.g. McCrone *et al*., 2005). As for children who need one-to-one early intervention services, they may benefit from telehealth services by qualified professionals who reside both within and outside Lebanon. While there are a handful of studies discussing telehealth in Lebanon (e.g., Abi Ramia *et al*., 2018; Shehadeh *et al*., 2020), the majority of the literature focuses on psychiatric disorders among adults and/or refugees, with no empirical work examining early intervention services for children with developmental disabilities. While it is possible for professionals residing abroad to offer such services via telehealth at reduced and affordable costs, the ultimate goal would be to train professionals in Lebanon to support community-wide and one-to-one interventions. This will ensure continuity of care within the country if and when professionals residing abroad can no longer offer such services. Furthermore, training professionals in Lebanon will ensure culturally appropriate practices. One possible avenue is offering certifications in early intervention for individuals who hold bachelors and master's degrees in a related field (e.g. psychology, speech language pathology, social work). It is noteworthy that the logistics of such services may prove complicated given the current state of healthcare systems in Lebanon, which will be discussed next.

### The Lebanese health care system and licensure considerations

The Lebanese mental health profession and its organization are still relatively new. The National Mental Health Program (NMHP) was launched in May 2014 within the Ministry of Public Health (MoPH) with the support of the World Health Organization (WHO), UNICEF, and International Medical Corps (IMC; El Chammay and Roberts, 2020). Even though several laws and regulations have since been published attempting to regulate the mental health profession in Lebanon, much work remains to be done. The mental health care system in Lebanon (for adults and children) does not have the required infrastructure nor does it have sufficient human or financial resources to respond to the needs of the Lebanese population adequately (El Chammay and Roberts, 2020). An analysis conducted by the MoPH (2016) sheds light on some of these gaps, including insufficient psychiatric beds, supplies, and a shortage of mental health professionals. It is worth noting that these findings were published in 2016. At the time of this writing, in early 2022, in the midst of what has been dubbed as one of the most severe global crises by the Word Bank (World Bank Group, 2021), the mental health gaps identified in 2016 are likely much worse today.

In a similar vein, most governmental mental health programs and support (when available) seem to be offered to refugees, low-income people, port blast victims, and individuals suffering from alcohol disorders with few or none aimed at children with developmental delays and disorders. In addition to the Lebanese MoPH, the Lebanese Ministry of Social Affairs (MSA) is tasked with providing information and support to individuals with developmental delays and disabilities. A look at the MSA's website highlights the harsh reality Lebanon is currently facing: The information is difficult to locate and what services are available and where to access them is far from clear. For a parent of a child with developmental delays and disorders, navigating Lebanon's health and social systems seems confusing and painful at best.

As for the financial coverage of mental health services, approximately half of the population in Lebanon is insured by private companies (Yehia *et al*., 2014; MoPH, 2015). Most of these private health insurance plans (especially the Lebanese ones) do not cover mental health services. Some Lebanese have health insurance through a workplace pension for public sector employees, such as the National Social Security Fund, the Army and Internal Security Forces, and the Public Servants. These workplace pensions cover, to varying degrees, psychiatric visits, psychotropic drugs, and inpatient care (Yehia *et al*., 2014; MoPH, 2015). To the best of our knowledge, these health coverages do not extend to early intervention services for children. Parents seeking such services need to cover these costs out of pocket. To make matters worse, charges from service providers keep increasing (in most cases) due to the devaluation of the salaries of mental health professionals. Lastly, to the best of our knowledge, most telehealth services are not covered by either public or private insurance companies in Lebanon, especially the ones pertaining to early intervention services, underscoring the inequitable access to early intervention services in the country.

Another potential problem that may arise is licensing. Attempts to create licensing rules and regulations for practicing psychologists in Lebanon remain relatively new (Lebanese University, 2017). Under the current National Mental Health Programme (NMHP) guidelines, to practice on Lebanese soil, one needs to be a Lebanese citizen. However, exceptions are made when the need for a certain expertise is high and difficult to find. This regulation also does not apply to Applied Behavior Analysis (ABA) therapists, nor does it apply to various professionals (e.g. speech-language pathologists, psychomotor therapists) that are often called to work with children with developmental delays and disorders. To the best of our knowledge, regulations concerning the use of telehealth in Lebanon could use more formalization and guidance. For more information regarding guidelines and considerations when offering telehealth services in Lebanon, please see Naal and *et al*. (2021). As authorities create licensing guidelines, it is imperative for them to take into account the current exodus of professionals in the field and ease the guidelines for foreigners willing to train and support the citizens who remain in the country. Additionally, offering regulations for the efficacious and ethical use of telehealth is an important next step.

In sum, even though some efforts have been made to try to move the field of psychology forward in Lebanon, much work remains to be done. The recent pandemic and socio-economic crisis the country has been grappling with has only exacerbated existing issues and hindered progress. According to Médecins Sans Frontières (MSF, 2021), the Lebanese health care system is disintegrating.

## Parental training

Parental training is another option to help supplement costly and often inaccessible professional interventions. Given the complexity of some developmental disorders and to bypass the lack of available professionals in Lebanon, parental training could be offered by professionals online (e.g. Simacek *et al*., 2020). Such training could provide parents with the skills and intervention techniques to support their children through such difficult times. Relatedly, some high-income countries such as Canada and the US offer online asynchronous parental and professional training to parents of children with developmental delays and disabilities. These services could be provided to parents of children with developmental disorders residing in various parts of Lebanon for no fee or a reduced fee.

Given the gravity of the situation in Lebanon and the importance of early intervention, we encourage professionals residing in high-income nations to extend such training and certification services to Lebanese professionals, or professionals-to-be. Professionals residing abroad may also offer services at reduced fees; however, it is critical for professionals offering services to adopt a culturally humble perspective to ensure adequate and appropriate care (see Fisher-Borne *et al*., 2015 for a discussion on cultural humility).

## The role of international NGOs

Lastly, NGOs and internationally funded centers are another avenue worth exploring. The Lebanese crisis has resulted in a proliferation of NGOs receiving funding from international sources (e.g. The Lebanese Food Bank, Bassma Lebanon, etc.). Amongst the few NGOs and centers left standing, a lack of funding and trained professionals is noted. Due to the pandemic and the socio-economic crisis, a large number of NGOs in Lebanon have now shut their doors. Those remaining are on life support. Financial assistance received by NGOs in Lebanon has mostly been directed toward the port blast victims and families grappling with extreme poverty (e.g. the Borgen Project, 2020). NGOs and centers offering early intervention services to children with developmental delays and disorders in Lebanon are very limited (e.g. The Lebanese Autism Society). Children with developmental delays and disorders have, once again, been relegated to the background. The need to offer both financial and (qualified) professional support for such early intervention programs is of utmost importance, as this may drastically change the future of the 2.1 million children currently living in Lebanon.

## Conclusion

Over the past 2 years, everyone residing in Lebanon has suffered tremendous loss and pain. That said, some parts of the population, such as children with developmental delays, are more vulnerable than others (Wallace and Rogers, 2010). These children have a window of time where intervention is likely to reap the greatest benefits. We should not let these children suffer for the rest of their lives because of a lack of decisive action by the international community.
